# Doctors and Insurance

**Published:** 1912-10-26

**Authors:** 


### DOCTORS AND INSURANCE.

### Remuneration Increased to 8s. 6d. per Member.

House of Commons, October 23, 1912.

The Speaker took the Chair at 2.50 p.m.

Sir Philip Magnus asked the Chancellor of the Exchequer whether he would now state what additional sum he was prepared to recommend Parliament to set aside for the provision of medical benefit under the National Insurance Act; and, if so, whether he could state what would be the normal amount offered for each insured person, with or without drugs, and what arrangements had been suggested for securing where necessary exceptional medical or surgical treatment.

Mr. Lloyd George, in reply, said that after careful consideration and after consultation with the members of the Advisory Committee, the Government had decided to increase the sum available for medical benefit to an amount which they were satisfied would secure full improved medical service for the whole population and at the same time afford adequate remuneration to medical officers required to do the work under the Act.

The House would remember that on the second reading of the Bill he stated that there was a margin which would enable him to increase the amount to be paid the doctors if a case were made out for such an increase. During the passage of the Bill provisions were inserted extending the cost of medical benefit. This and other charges at the same time absorbed the margin available for those purposes over and above the 6s. assumed in the estimates.

The Government had always recognised that the duties devolving upon these societies would necessitate additional contributions from the Exchequer. At the same time, the Government recognised the reasonableness of the demand of the medical profession for a guaranteed or minimum rate of remuneration. The financial proposals of the Government might be briefly summarised as follows:

It was proposed to add to the amount available for medical benefit a further sum equivalent of 2s. 6d. per insured person per annum.

This would enable the provision made for medical benefit to be raised to 8s. 6d. per head of insured persons—(cheers)—with a small additional sum to be reserved.

The demand of the British Medical Association exceeded 12s.

Of this sum of 8s. 6d., it was proposed that a minimum of 6s. 6d. should be denfiitely assigned as medical remuneration, 1s. 6d. would be for drugs, and a further 6d. would be available for drugs where required. Otherwise it would be available for medical remuneration.

Counting the further sum for sanatorium benefit for the treatment of tuberculosis, the total remuneration the doctors would receive, inclusive of drugs, would amount to 7s., or possibly 7s. 6d.

This additional grant would involve a total annual increased cost of £1,650,000, which would be made under such conditions as would secure the enhanced efficiency of the medical service for the industrial population, and the doctors would have to give such certificates as were necessary for sickness and disablement, and would have to keep such a simple record of diseases as was requisite. He was publishing at once a full statement of the effect of these proposals.

Replying to Mr. Ward, the Chancellor of the Exchequer said the 3s. which it was stated during the debate on the Bill would be available for additional benefits would now be taken by the doctors.
—*Westminster Gazette.*

